# Who drives weight stigma? A multinational exploration of clustering characteristics behind weight bias against preconception, pregnant, and postpartum women

**DOI:** 10.1038/s41366-025-01725-5

**Published:** 2025-01-28

**Authors:** Haimanot Hailu, Angela C. Incollingo Rodriguez, Anthony Rodriguez, Helen Skouteris, Briony Hill

**Affiliations:** 1 Health and Social Care Unit, School of Public Health and Preventive Medicine, Monash University, Melbourne, VIC Australia; 2 Psychological & Cognitive Sciences, Department of Social Science & Policy Studies, Worcester Polytechnic Institute, Worcester, MA USA; 3 RAND, Boston, MA USA; 4 Warwick Business School, The University of Warwick, Coventry, UK

**Keywords:** Obesity, Public health

## Abstract

**Background:**

Weight bias is a global health challenge and community members are endorsed as the most common source of weight bias. The nature of weight biases specifically against preconception, pregnant, and postpartum (PPP) women from the perspective of community members is not known, especially in terms of cross-cultural trends. We investigated the magnitude of explicit and implicit weight bias and profiles of characteristics associated with harbouring weight bias.

**Methods:**

We conducted a multinational investigation of clusters of factors associated with weight bias against PPP women (May–July 2023). Community members from Australia, Canada, United States (US), United Kingdom (UK), Malaysia, and India completed a cross-sectional survey measuring explicit and implicit weight biases, beliefs about weight controllability, and awareness of sociocultural body ideals. Hierarchical multiple regression and latent profile analyses identified clusters of factors associated with weight bias.

**Results:**

Participants from India reported the lowest explicit weight bias (B = −0.45, *p* = 0.02). Participants from Australia (B = −0.14, *p* = 0.04) and the UK (B = −0.16, *p* = 0.02) (vs. US) reported the lowest implicit weight bias. Three distinct profiles were identified clustering on body mass index (BMI) and weight-controllability beliefs: *low-BMI/moderate-beliefs, high-BMI/more biased beliefs*, and *high-BMI/less biased beliefs*. Profile membership varied by country of residence and weight bias outcomes with *low-BMI/moderate-beliefs* profiles containing more people from non-Western countries and with low explicit weight bias.

**Conclusions:**

Explicit and implicit weight bias was harboured by participants across all included nations, although less pronounced in non-Western countries. Our profiles highlight that individuals who held a stronger belief that weight is controllable, regardless of their body weight, should be targeted for interventions to eliminate weight stigma.

## Background

Weight bias or weight stigma is a global problem precipitating numerous consequences for overall health and wellbeing [1, 2]. Referring to negative beliefs, attitudes, assumptions, and judgments about people living with overweight and obesity, weight bias can be intentional and conscious (explicit) or unintentional and covert (implicit) [3]. Weight bias is experienced more often among women than men, with women reporting, on average, three stigmatising experiences daily [4]. Preconception, pregnant, and postpartum (PPP) women are a priority victim of stigmatisation due to societal norms and expectations of slimness [5] and the susceptibility of weight gain during reproductive periods [6]. About 70% of pregnant or postpartum women living with obesity face weight bias from at least one source including family, friends, partners, work, media, education, healthcare providers, the community, and even strangers [7]. This multi-setting exposure to weight bias over the childbearing period increases women’s vulnerability to the consequences of weight bias [7, 8], which during the PPP period includes lower uptake of reproductive healthcare services, poor mental and behavioural health, further weight gain, stress, and long-term undesired maternal health outcomes with intergenerational consequences [6, 9]. Thus, understanding and preventing weight stigma in the PPP period is essential to promoting maternal-child health and wellbeing.

While the experiences of weight bias from the victim’s perspective are well documented, fully addressing weight bias requires also understanding it from the perpetrator’s perspective [6, 10, 11]. This highlights a gap in our knowledge about the nature of weight bias and the characteristics of individuals who harbour weight bias in society [6, 10]. Addressing these gaps is important as (1) weight bias is pervasive in society and often normalised [12]; (2) the general public is endorsed as the most common source of weight bias by pregnant and postpartum women [7]; and (3) societal attitudes and cultural norms shape individual attitudes and behaviours leading to weight bias pervasiveness [5, 12].

The broader weight stigma literature, which predominantly includes samples from Western countries (e.g. USA, Australia, and Canada) indicates the pervasiveness of weight bias in these nations [2, 13]. However, emerging evidence suggests that weight bias is also recognised in non-Western countries and is becoming a global challenge [1]. Previous studies provided clues as to which beliefs and sociocultural standards might precipitate weight stigma. For instance, attributing obesity to individual behaviours such as nutrition and physical activity is associated with increased weight bias even though experimental studies targeting this attribution report mixed findings [14]. Similarly, in studies that assessed weight bias against people with obesity in general, the association of sociodemographic factors with weight bias varied [2, 15]. Also, self-perception of living with obesity and empathy both appear to be associated with lower weight bias [16, 17]. However, this evidence hampers our understanding of how to intervene against weight bias targeting women internationally because it comes mainly from Western countries and it provides no insight about the potential beliefs and societal norms that may explain the development of weight biases against PPP women. The presence of extra pressure for women to conform to societal body ideals [18] suggests that the nature of weight bias may differ from weight bias against the general population. However, to our knowledge, no studies have compared these trends in weight bias against PPP women between people from of Western and non-Western countries.

Given the dearth of knowledge on the nature of weight bias towards PPP women and the characteristics of individuals who harbour weight bias in society, we conducted a multinational assessment of weight bias to: (1) investigate the magnitude of explicit and implicit weight bias, and attitudes towards PPP women with overweight or obesity across Western and non-Western contexts; and (2) identify and profile clusters of characteristics associated with weight bias, ultimately allowing us to explore “who drives weight stigma?”

## Methods

### Study design and participants

Participants were recruited via Qualtrics panels, social media, and public advertisements and completed an online survey on Qualtrics (Qualtrics, Provo, UT) between May and July 2023. We recruited a cross-sectional sample of community members (i.e., members of the public) aged 18 years or over and residing in Australia, Canada, United States (US), United Kingdom (UK), Malaysia, and India. Western countries (i.e., Australia, Canada, UK, and US) were selected because they have a comparable and high magnitude of obesity (as measured by body mass index [BMI]) and similar sociocultural values of thinness [2]. Malaysia and India (non-Western countries) are among the countries with an increasing rate of overweight and obesity [19, 20]. Individuals who were unable to read English were excluded.

### Ethics approval and consent to participate

All methods were performed in accordance with the relevant guidelines and regulations, and reported per the Strengthening the Reporting of Observational Studies in Epidemiology (STROBE) guideline (Supplementary Table S1). The study was approved by the Monash University Human Research Ethics Committee (36876). All participants who accessed the survey link on the recruitment materials were invited to read the electronic explanatory statement and consent was indicated by continuation to the online survey.

### Procedure and measures

Participants completed self-report measures of sociodemographic characteristics, explicit and implicit weight bias, as well as their beliefs about, and attitudes towards PPP women living with overweight or obesity. The measures were modified to specifically refer to ‘PPP women’ instead of ‘people’ with obesity. The main outcomes were explicit weight bias, measured using the Fat Phobia Scale (FPS) [21]; implicit weight bias, assessed using the Weight Implicit Association Test (IAT) [22]; and attitude towards PPP women, measured using the Attitudes Towards People with Obesity (ATOP) tool [23]. The associated factors included beliefs about the controllability of obesity (Beliefs About Persons Living with Obesity Scale; BAOP) [23]; societal norms (Awareness sub-scale of the Sociocultural Attitudes Toward Appearance Questionnaire; SATAQ) [18]; and Empathy (Empathy subscale of the Fat Attitudes Assessment Toolkit; FAAT) [24]. Higher scores on each measure indicate higher weight bias, less stigmatising attitudes, less biased belief, more awareness of societal norms, and greater empathy. A detailed text description of the measures is provided in Supplementary File Section II.

### Sample size and data analysis

A sample size of 483 would provide 80% power to detect small effect sizes and the association between outcome and associated factors [15]. Descriptive and regression analyses were performed in SPSS 29.0 [25]. Data were missing for age (missing *n* = 53), weight (*n* = 12), height (*n* = 8), and IAT score (*n* = 7) and were missing at random (χ^2^ = 15.037, DF = 18, *p* = 0.66) [26], addressed by multiple imputation. One-way ANOVAs tested for differences in characteristics across country, with post-hoc Tukey HSD tests probing specific contrasts. Univariable linear regressions explored the associations between bias/attitude scores (FPS, ATOP, and IAT) and participant characteristics, BAOP, empathy, and SATAQ, and therefore identified candidate variables for inclusion in multiple regression analyses; variables with statistically significant associations (*p* < 0.05) were retained. Hierarchical multiple regressions examined potential factors associated with FPS, ATOP, and IAT scores: Step 1 contained demographic variables; Step 2 introduced BMI and weight self-perception; Steps 3 and 4 introduced BAOP and SATAQ, respectively.

#### Latent profile analysis

Using Mplus [27], latent profile analysis across BMI, BAOP, SATAQ, and weight self-perception disentangled sample heterogeneity and identity subgroups. Model fit was determined across the following: −2 Log-Likelihood (−2LL), Akaike Information Criterion (AIC), Bayesian Information Criterion (BIC), sample size adjusted Bayesian Information Criterion (aBIC), Vuong-Lo- Mendell-Rubin Likelihood Ratio Test (VLMR), Lo-Mendell-Rubin Test (LMRT), and Bootstrapped Likelihood Ratio Test (BLRT). A solution with lower −2LL, AIC, BIC, and aBIC represents a better fit. The VLMR, LMRT, and BLRT statistically test whether a given solution (*k*) is an improvement over a *k* – 1 solution (e.g., four vs. three profiles) [28]. Once the most plausible solution was identified, we used the 3-step approach [29] wherein logits for classification probabilities for the most likely latent class membership are used for classification which accounts for measurement error. Using logits rather than modal assignment, emergent profiles were compared on several covariates (e.g., residence, gender, ethnicity) and distal outcomes (e.g., explicit weight bias – fat phobia, attitude toward people with obesity) using the Bolck, Croon, and Hagenaars (BCH) approach [30] method for continuous variables and Distal Categorical outcome (DCAT) [27] function for categorical variables. This approach allowed for identifying clusters of characteristics associated with weight bias (given the heterogeneity across each outcome variable during regression analysis) giving insight into actionable clusters for weight stigma prevention.

## Results

### Participant characteristics

Table 1 shows participant characteristics. Of 683 participants recruited, 146 did not complete any survey items and 23 failed attention check questions and were removed, leaving a final sample of 514. Mean (M) age was 49 years (standard deviation (SD) 17.7). The majority of the sample were female (*n* = 316; 61.5%) and White (*n* = 310; 60.1%). Overall, 47.2% of the participants reported a BMI of 25 kg/m^2^ or above, which was associated with country of residence (χ^2^ = 51.4; *p* < 0.001); a greater proportion of participants from the US had a BMI of ≥30 kg/m^2^ compared to other countries.

**Table 1:** Participant demographic characteristics and difference across country of residence.

Variable	Overall (*N* = 514)	Australia (*N* = 110)	Canada (*N* = 82)	USA (*N* = 80)	UK (*N* = 81)	Malaysia (*N* = 79)	India (*N* = 82)	*p*-value
Age, mean (SD)	49.0 (17.7)	59.1 (16.1)	54.1 (14.1)	59.8 (17.6)	46.8 (15.7)	37.9 (10.8)	32.9 (10.5)	<0.001^b^
Gender								
Male	196 (38.1%)	39 (35.5%)	33 (40.2%)	18 (22.5%)	24 (29.6%)	40 (50.6%)	42 (51.2%)	
Female	316 (61.5%)	71 (64.5%)	49 (59.7%)	61 (76.25%)	56 (69.1%)	39 (49.4%)	40 (48.8%)	
Non-binary	2 (0.4%)	–	–	1 (1.3%)	1 (1.2%)	–	–	
Marital status								<0.001^c^
Single	161 (31.3%)	24 (21.8%)	28 (34.1%)	17 (21.3%)	34 (42%)	28 (35.4%)	30 (36.6%)	
Married	267 (51.9%)	54 (49.1%)	43 (52.4%)	36 (45%)	34 (42%)	48 (60.8%)	52 (63.4%)	
Divorced/widowed	84 (16.3%)	32 (29.1%)	10 (12.2%)	26 (32.5%)	13 (16%)	3 (3.8%)	–	
Prefer not to answer	2 (0.4%)	–	1 (1.2%)	1 (1.3%)	–	–	–	
Educational status								< 0.001^c^
High school or less	121 (23.5%)	38 (34.5%)	10 (12.2%)	21 (26.3%)	30 (37%)	20 (25.3%)	2 (2.4%)	
TVET	109 (21.2%)	31 (28.2%)	35 (42.7%)	32 (40%)	16 (19.8%)	10 (12.7%)	4 (4.9%)	
BSc or Associate degree	189 (36.8%)	28 (25.5%)	33 (40.2%)	32 (40%)	19 (23.5%)	44 (55.7%)	33 (40.2%)	
Master’s degree and above	95 (18.5%)	13 (11.8%)	4 (4.9%)	14 (17.5%)	16 (19.8%)	5 (6.3%)	43 (52.4%)	
Ethnicity								<0.001^c^
Asian	172 (33.5%)	2 (1.8%)	12 (14.6%)	3 (3.8%)	3 (3.7%)	76 (96.2%)	76 (92.7%)	
White	310 (60.3%)	100 (90.9%)	66 (80.5%)	69 (86.3%)	72 (88.9%)	2 (2.5%)	1 (1.2%)	
Others^a^	32 (6.2%)	8 (7.3%)	4 (4.9%)	8 (10%)	6 (7.4%)	1 (1.3%)	5 (6.1%)	
Employment								<0.001^c^
Yes	274 (53.3%)	39 (35.5%)	38 (46.3%)	21 (26.3%)	43 (53.1%)	62 (78.5%)	71 (86.6%)	
No	233 (45.3%)	68 (61.8%)	42 (51.2%)	59 (73.8%)	37 (45.7%)	16 (20.3%)	11 (13.4%)	
Not provided	7 (1.4%)	3 (2.7%)	2 (2.4%)	–	1 (1.2%)	1 (1.3%)	–	
BMI (kg/m^2^)								<0.001^c^
Below 18.5	31 (6%)	4 (3.6%)	3 (3.7%)	6 (7.5%)	3 (3.7%)	10 (12.7%)	05 (6.1%)	
18.5 to <25	240 (46.7%)	38 (34.5%)	35 (42.7%)	28 (35%)	38 (46.9%)	47 (59.5%)	54 (65.9%)	
25 to <30	140 (27.2%)	40 (36.4%)	27 (32.9%)	20 (25%)	23 (28.4%)	12 (15.2%)	18 (22%)	
30 or higher	103 (20%)	28 (25.5%)	17 (20.7%)	26 (32.5%)	17 (21%)	10 (12.7%)	5 (6.1%)	
Self-Perception of living with overweight or obesity								–
Yes	181 (35.2%)	45 (40.9%)	27 (32.9%)	34 (42.5%)	34 (42%)	22 (27.8%)	19 (23.2%)	
No	331 (64.4%)	65 (59.1%)	55 (67.1%)	44 (55%)	47 (58%)	57 (72.2%)	63 (76.8%)	
Not provided	2 (0.4%)	–	–	2 (2.5%)	–	–	–	
Family member living with overweight or obesity								–
Yes	229 (44.6%)	46 (41.8%)	29 (35.4%)	39 (48.8%)	38 (46.9%)	34 (43%)	43 (52.4%)	
No	282 (54.9%)	64 (58.2%)	53 (64.6%)	39 (48.8%)	43 (53.1%)	44 (55.7%)	39 (47.6%)	
Not provided	3 (0.6%)	–	–	2 (2.5%)	–	1 (1.3%)	–	
Friend living with overweight or obesity								–
Yes	274 (53.3%)	56 (50.9%)	39 (47.6%)	38 (47.5%)	33 (40.7%)	53 (67.1%)	55 (67.1%)	
No	237 (46.1%)	54 (49.1%)	43 (52.4%)	41 (51.2%)	47 (58%)	25 (31.6%)	27 (32.9%)	
Not provided	3 (0.6%)	–	–	1 (1.3%)	1 (1.2%)	1 (1.3%)	–	

*p*-value not provided for variables for which chi-square assumptions were not fulfilled.

^a^Black, multiracial, Hispanic, Indigenous, Middle Eastern; *TVET* Technical Vocational Education and Training.

^b^Post hoc.

^c^Chi squared test.

### Magnitude of weight bias

Supplementary Tables S2 and S3 present descriptive information and correlations regarding weight bias and related measures. Fat phobia, ATOP, and IAT scores differed by country of residence but not gender. The BAOP, empathy, and SATAQ scores were not statistically significantly different by country of residence. Empathy scores differed by gender. There was no statistically significant correlation between FPS and IAT.

### Factors associated with explicit weight bias

The final model to predict FPS (fat phobia; model 4) was statistically significant (R^2^ = 0.22, F = 7.625, *p* < 0.001), explaining 22% of the variance in FPS scores. Scores differed by country of residence and employment status, explaining 17% of variance in FPS. Residents of Australia, Canada, and the UK reported the highest FPS scores, while the residents of India (B = −0.45, *p* = 0.02) reported the lowest. After accounting for demographics, BMI, and weight self-perception, a stronger belief that weight is not controllable (less biased) was associated with lower FPS scores (B = −0.13, *p* < 0.01) while a greater awareness of sociocultural standards of body ideals (more biased) was associated with higher FPS scores (B = 0.03, *p* < 0.01; Supplementary Table S4).

### Factors associated with attitudes towards PPP women living with overweight or obesity

The full model to predict ATOP (model 4) was statistically significant (R^2^ = 0.29, F = 7.625, *p* < .001) explaining 29% of the variance in ATOP scores (Supplementary Table S5). Older people (B = 0.09, *p* = 0.04) and individuals with higher BMI (B = 0.30, *p* = 0.02) held less stigmatising attitudes. After accounting for demographic variables, BMI, and weight self-perception, a stronger belief that weight is not controllable (less biased) was associated with less stigmatising attitudes (B = 0.73, *p* < 0.01) while a greater awareness of sociocultural standards of body ideals (B = −1.20, *p* < 0.01) was associated with more stigmatising attitudes. BAOP and SATAQ scores explained 14% and 8% of the variance in ATOP score, respectively.

### Factors associated with implicit weight bias

The full model to predict implicit weight bias (model 2) was statistically significant (R^2^ = 0.14, F = 5.43, *p* < 0.001). Multivariable analyses (Supplementary Table S6) revealed that IAT scores differed by country of residence with residents of Australia (B = −0.14, *p* = 0.04) and the UK (B = −0.16, *p* = 0.02) reporting the lowest scores, but no statistically significant difference was observed for Malaysia (*p* = 0.67) and India (*p* = 0.67). Individuals with higher educational status (B = −0.20, *p* < 0.01) exhibited lower implicit weight bias. Older people (B = 0.01, *p* < 0.01) and individuals with White ethnicity (B = 0.18, *p* = 0.04) exhibited higher implicit weight bias, explaining 13% of the variance in IAT score. A stronger belief that weight is not controllable (less biased) (B = −0.01, *p* = 0.01) was associated with lower implicit weight bias.

### Latent profiles

Results from the latent profile analysis (LPA) analyses are presented in Supplementary Table S7. A series of 4 profile models were estimated and fit indices evaluated. All of the information criteria (-2LL, AIC, BIC, and aBIC) continued to decrease through the 4-profile solution, except for BIC which increased for the 4-profile model – indicating the 3-profile solution was a better model. That said, likelihood ratio tests initially indicated that 2 profile solution was a better fit than the 3-profile solution (VLMR: *p* = 0.12, LMR: *p* = 0.12); however, the BLRT indicated that 3 profiles were better than 2 profiles. When inconsistencies in likelihood ratio tests arise, the recommendation is to use BIC to make final determinations [28]. As such, the 3-profile solution was retained. The three groups differed primarily in terms of average BMI, BAOP scores, and self perception of living with obesity. SATAQ scores were generally comparable across groups (range: 3.2–3.442).

*Profile 1* (*n* = 326, 63.4%) – we labelled this profile *‘low-BMI and moderate-beliefs’*. It was characterised by individuals with an average BMI of 22.89 kg/m^2^ (SE = 0.20), moderate BAOP (M = 16.11, SE = 0.39), and zero individuals self-perceived to be living with obesity. *Profile 2* (*n* = 110, 21.4%) – we labelled this profile *‘high-BMI and more biased beliefs’*. This profile was characterised by individuals with an average BMI of 30.26 kg/m^2^ (SE = 0.97), low BAOP (M = 13.77, SE = 0.84), and 86% of individuals self-perceived to be living with overweight or obesity. *Profile 3* (*n* = 78, 15.2%) – was labelled *‘high-BMI and less biased beliefs’*. It was characterised by individuals with an average BMI of 30.33 kg/m^2^ (SE = 0.89), the highest BAOP (21.66, SE = 2.55), and 100% of individuals self-perceived to be living with overweight or obesity.

#### Differences on sociodemographic characteristics

**Fig. 1:**
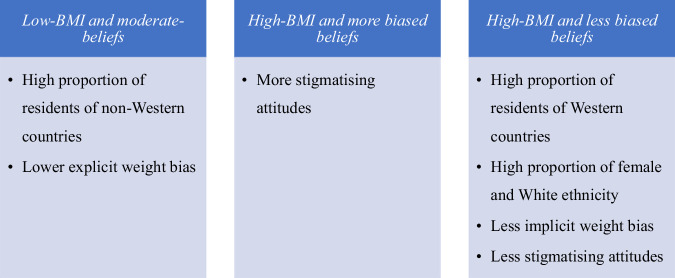
Summary of latent profile analyses incorporating sociodemographic and weight bias measures across three distinct profiles.

Profile descriptives for sociodemographic characteristics and dependent variables are presented in Table 2 and Fig. 1. Figure 1 is provided to support the interpretation of the findings. Individuals with *high-BMI and less biased beliefs*, compared to *low-BMI and moderate-beliefs*, were more likely to be female (81% vs 56%), White (76% vs 54%), and living in Western countries (Australia: 26% vs 20%, USA: 19% vs 13%, UK: 21% vs 14%). With regard to education, individuals with *high-BMI and more biased beliefs*, compared to *low-BMI and moderate-beliefs*, were marginally more likely to have technical or vocational training (33% vs 21%) and less likely to have a master’s degree and above (8% vs 21%). There were no differences in age, employment, or marital status.

**Table 2:** Means, standard errors, and statistical results for latent profile analyses.

Variable	Profile 1	Profile 2	Profile 3	Comparisons
Age	47.94 (1.03)	51.47 (2.58)	50.27 (2.35)	χ^2^ = 3.133, *p* = 0.209
Gender				1 v 3: χ^2^ = 17.9, *p* < 0.001***
Residence				1 v 3, χ^2^ = 19.9, *p* < 0.001^***^
Australia	0.20 (0.02)	0.22 (0.06)	0.26 (0.06)	
Canada	0.17 (0.02)	0.11 (0.05)	0.18 (0.05)	
USA	0.13 (0.02)	0.22 (0.06)	0.19 (0.06)	
UK	0.14 (0.02)	0.17 (0.06)	0.21 (0.06)	
Malaysia	0.17 (0.02)	0.11 (0.05)	0.13 (0.05)	
India	0.19 (0.02)	0.17 (0.05)	0.03 (0.03)	
Ethnicity				1 v 3: χ^2^ = 10.9, *p* = 0.004^**^
Asian	0.40 (0.03)	0.26 (0.06)	0.29 (0.06)	
White	0.54 (0.03)	0.64 (0.07)	0.76 (0.06)	
Others*	0.06 (0.01)	0.10 (0.04)	0.04 (0.03)	
Education				1 v 2: χ^2^ = 6.3, *p* = 0.099*
Highschool or less	0.22 (0.02)	0.22 (0.06)	0.29 (0.07)	
Technical or vocational training	0.21 (0.02)	0.32 (0.07)	0.11 (0.05)	
Bachelor or Associate degree	0.36 (0.03)	0.39 (0.07)	0.37 (0.07)	
Master’s degree and above	0.21 (0.02)	0.08 (0.05)	0.23 (0.06)	
Employment				χ^2^ = 6.30, *p* = *0.18*
Yes	0.58 (0.03)	0.42 (0.07)	0.51 (0.07)	
No	0.41 (0.03)	0.58 (0.07)	0.46 (0.07)	
Not provided	0.01 (0.01)	0.00 (0.02)	0.03 (0.02)	
Marital status				χ^2^ = 7.14, *p* = *0.31*
Single	0.33 (0.03)	0.30 (0.07)	0.28 (0.07)	
Married	0.54 (0.03)	0.47 (0.07)	0.52 (0.07)	
Divorced/widowed	0.132 (0.02)	0.23 (0.06)	0.20 (0.06)	
Prefer not to answer	0.00 (0.00)	0.01 (0.01)	0.00 (0.00)	
FPS	2.85 (0.05)	3.16 (0.12)	2.90 (0.09)	1 v 2: χ^2^ = 5.3, *p* = 0.02**
ATOP	60.82 (0.87)	52.60 (6.04)	86.52 (15.04)	1 v 3: χ^2^ = 3.0, *p* = 0.08*
				2 v 3: χ^2^ = 2.7, *p* = 0.09*
IAT	0.55 (0.03)	0.51 (0.07)	0.43 (0.06)	1 v 3 = χ^2^ = 3.0, *p* = 0.08*

Others: Black, Multiracial, Hispanic, Indigenous, Middle Eastern.

Small group removed for gender: Nonbinary (*n* = 2).

***P < 0.001; **P < 0.05; *P ≤ 0.09.

#### Outcomes

Compared to *low-BMI and moderate-beliefs*, individuals with *high-BMI and more biased beliefs* had significantly higher FPS scores (i.e. more explicit weight bias). Individuals with *high-BMI and less biased beliefs* had marginally lower IAT scores (i.e. lower implicit weight bias) compared to those with *low-BMI and moderate-beliefs*. Additionally, individuals with *high-BMI and less biased beliefs* had marginally higher ATOP scores (i.e. less stigmatising attitudes) compared to *low-BMI and moderate-beliefs* and *high-BMI and more biased beliefs*.

Generally, the profile of *low-BMI, moderate-beliefs* compared to *High-BMI, more biased beliefs* and *High BMI, less biased beliefs* was characterised by lower explicit weight bias and constitutes a high proportion of participants from developing countries. *High-BMI, less biased belief*, on the other hand, constitutes high proportion of female, White ethnic group and participants from Western countries. Also, this profile was characterised by low implicit weight bias and less stigmatising attitudes compared to either *low-BMI, moderate beliefs* or *High-BMI, more biased beliefs* groups (Fig. 1).

## Discussion

We are the first to investigate weight bias against PPP women among community members, and to identify unique profiles demonstrating clusters of relevant factors. Furthermore, we achieve this using multinational data collected from six countries spanning Western and non-Western cultures. Explicit weight bias, stigmatising attitudes, and implicit weight bias against PPP women were evident in all countries with the former being higher in Western countries. We identified three mutually exclusive and homogenous profiles based on BMI, weight-self-perception, and controllability beliefs. Profiles with *‘high-BMI and less biased beliefs’* were characterised by less stigmatising attitudes, implicit weight bias, and higher proportions of White ethnicity, women, and people from Australia, USA and UK. Profiles with ‘*low-BMI and moderate-beliefs’* were characterised by lower explicit weight bias and the highest proportion of individuals from Non-western countries. We found a distinct relationship between controllability beliefs and weight bias. We identified differences in profile membership by gender, ethnicity, and country of residence, with a higher proportion of people from Asia, and people from non-Western countries in profiles lower in explicit weight bias.

The explicit and implicit weight bias and stigmatising attitudes towards PPP women across all the countries highlight an overarching negative societal view towards PPP women living in larger bodies. While unsurprising, we are the first to demonstrate these attitudes from the perpetrators’ perspectives indicating that community members may freely express negative attitudes or feelings about PPP women living with overweight or obesity, which aligns with broader social acceptability of weight stigma [12]. Here, this may contribute to the perpetual social narrative that women must be both functional, beautiful, and slim [5].

Our findings highlighted the importance of country of residence in weight bias formation with participants from Western countries reporting higher explicit bias. In some cultures, especially in non-Western countries (e.g., Arabic nations), higher weight is valued as attractive, whereas in Western countries, thinness is considered attractive [31]. It is well known that Western countries have a significant proportion of people migrating from different parts of the world [32]. Our findings suggest that those people tend to adopt Western thinness ideals and perhaps weight bias, regardless of their country or origin. It was interesting to note that across our three profiles, a comparable greater awareness of societal standards of body ideals was reported. Similarly, we found a greater awareness of societal standards of body ideals was associated with more explicit weight bias and more stigmatising attitudes. The influence of sociocultural appearance norms may be particularly important for PPP women due to the extra societal expectations and perpetuation of thinness ideals towards women [18]. Our study also highlighted the presence of weight bias and controllability beliefs in non-Western countries, albeit to a lesser extent. The preference for and consideration of thinness as a beauty ideal is becoming a norm in non-Western countries [33]. This is concerning because it often intersects with other social stigmas such socio-economic and demographic characteristics [34]. Despite a notable gap in research in this area, there is emerging evidence suggesting weight bias against pregnant and postpartum women is becoming a problem in low and middle-income countries [34]. Therefore, our findings have implicated an urgent need for interventions targeting weight bias against PPP women in Western countries while also highlighting the necessity of initiatives in non-Western countries.

The highest explicit weight bias was observed in those profiled with *high-BMI and more biased beliefs* compared to *low-BMI and moderate-beliefs*. We also observed a consistent general trend where people with *high-BMI and less biased beliefs* reported the highest positive attitudes and lower implicit weight bias against PPP women with obesity. Our findings underscore that controllability beliefs are key in the manifestation of weight bias regardless of BMI and weight self-perception; this was consistently demonstrated in all analyses. These beliefs are concerning because they often lead to stereotyping people living with obesity as lazy, less competent, undisciplined, and responsible for their higher weight, which then leads to weight bias [12]. Additionally, we found a higher proportion of women and White ethnicity in *high-BMI and less biased beliefs* profiles. However, people with higher BMI were profiled across both *high-BMI and more biased beliefs* and *high-BMI and less biased beliefs* suggesting that some people may be less likely to hold biases against their own in-group [16], while others may nonetheless perpetuate ingroup stigma. Therefore, it would be relevant for weight bias interventions to involve people living with obesity to either target the ingroup bias or as lived experience advocates. Involving people with lived experience helps in effectively translating knowledge into practice and ensures the relevance and feasibility of interventions [35].

The strengths of the current study include the inclusion of a large sample of community members and a wide range of demographic characteristics. Also, our study utilised multiple measures of weight bias which helped provide a comprehensive picture of the nature of weight bias towards PPP women. Limitations include that despite having a relatively large sample, nuanced comparisons were not possible due to statistical power, however, we were adequately powered to detect the minimum effect sizes and address our objectives. Reliability values for the SATAQ (0.05) and BAOP (0.66) were lower than anticipated, which may be an artefact of our sample’s cross-cultural nature. Nevertheless, item deletion analysis was performed to explore the impact of removing specific items on Cronbach’s alpha; no removal of a specific item increased the score.

Our robust and innovative study offers a comprehensive picture of weight bias against PPP women from perpetrators’ perspectives across Western and non-Western nations. By clustering characteristics into three distinct profiles, we offer insights into which community clusters might be prioritised in research and engaged in interventions. Namely, our profiles highlight that individuals who hold a stronger belief that weight is controllable should be prime intervention targets regardless of their BMI. Overall, this work provides previously lacking evidence and a novel framework to promote the development of successful weight stigma prevention strategies which will contribute to improved health outcomes for PPP women and their children. Future research should identify strategies to foster inclusive societies that embrace body diversity.

## Supplementary information

Supplemental file

## Data Availability

The data are not publicly available. De-identified participant data will be available upon formal request to principal investigator BH (email: briony. hill@monash.edu) to individuals for research purposes.
