# Supplementary material for: Who drives weight stigma? A multinational exploration of clustering characteristics behind weight bias against preconception, pregnant, and postpartum women
Source: Int J Obes (Lond). 2025 Jan 28;49(5):931–7. doi: 10.1038/s41366-025-01725-5 (PMC12095067; doi:10.1038/s41366-025-01725-5)
Supplement: Supplementary file 1 — Supplemental file [file 41366_2025_1725_MOESM1_ESM.docx]

**Supplementary File**

**Who drives weight stigma? A multinational exploration of clustering characteristics behind weight bias against preconception, pregnant, and postpartum women**

Haimanot Hailu, Angela C. Incollingo Rodriguez, Anthony Rodriguez, Helen Skouteris, Briony Hill

**Health and Social Care Unit, School of Public Health and Preventive Medicine, Monash University, Melbourne, Australia** (H Hailu MPH, Prof H Skouteris PhD, B Hill PhD); **Psychological & Cognitive Sciences, Department of Social Science & Policy Studies, Worcester Polytechnic Institute, Worcester, Massachusetts, USA** (AC Incollingo Rodriguez PhD); **RAND, Boston, Massachusetts, USA** (A Rodriguez PhD); **Warwick Business School, The University of Warwick, Coventry, UK** (Prof H Skouteris PhD)

Correspondence to:

Dr Briony Hill, Health and Social Care Unit, School of Public Health and Preventive Medicine, Monash University, 553 St Kilda Road, Melbourne VIC 3004, Australia

Email: briony.hill@monash.edu

Contents

[**Section I: STROBE checklist, descriptive and hierarchical regression tables** 1](#_Toc178181590)

[**Table S1: STROBE Statement—Checklist of items that should be included in reports of *cross-sectional studies*** 1](#_Toc178181591)

[**Table S2: Distribution of FPS, ATOP, IAT, BAOP, Empathy and SATAQ across countries of residence and gender** 3](#_Toc178181592)

[**Table S3: Correlations between FPS, IAT, ATOP, BAOP, Empathy and SATAQ** 4](#_Toc178181593)

[**Table S4: Hierarchical multiple regression of factors associated with explicit weight bias** 5](#_Toc178181594)

[**Table S5: Hierarchical multiple regression of factors associated with attitude towards PPP women living with overweight or obesity** 6](#_Toc178181595)

[**Table S6: Hierarchical multiple regression of factors associated with implicit weight bias** 7](#_Toc178181596)

[**Section II: Description of the measures** 8](#_Toc178181597)

[**Section III: Details of Latent profile analysis method** 10](#_Toc178181598)

[**Table S7. Model fit indices for Latent Profiles** 10](#_Toc178181599)

[**Section IV: Univariate analysis results** 11](#_Toc178181600)

[**Table S8: Univariate analysis of factors associated with explicit weight bias (FPS)** 11](#_Toc178181601)

[**Table S9: Univariate analysis of factors associated with attitude towards PPP living with overweight or obesity (ATOP)** 12](#_Toc178181602)

[**Table S10: Univariate analysis of factors associated with implicit weight bias (IAT)** 13](#_Toc178181603)

[**References** 14](#_Toc178181604)

# **Section I: STROBE (Strengthening the Reporting of Observational Studies in Epidemiology) checklist, descriptive and hierarchical regression tables**

## **Table S1: STROBE Statement—Checklist of items that should be included in reports of *cross-sectional studies***

|  | Item No | Recommendation | Page No |
| --- | --- | --- | --- |
| **Title and abstract** | 1 | (*a*) Indicate the study’s design with a commonly used term in the title or the abstract | Title |
|  |  | (*b*) Provide in the abstract an informative and balanced summary of what was done and what was found | Abstract |
| Introduction | | | |
| Background/rationale | 2 | Explain the scientific background and rationale for the investigation being reported | 1-2 |
| Objectives | 3 | State specific objectives, including any prespecified hypotheses | 2-3 |
| Methods | | | |
| Study design | 4 | Present key elements of study design early in the paper | 3 |
| Setting | 5 | Describe the setting, locations, and relevant dates, including periods of recruitment, exposure, follow-up, and data collection | 3-4 |
| Participants | 6 | (*a*) Give the eligibility criteria, and the sources and methods of selection of participants | 3 |
| Variables | 7 | Clearly define all outcomes, exposures, predictors, potential confounders, and effect modifiers. Give diagnostic criteria, if applicable | 4 |
| Data sources/ measurement | 8* | For each variable of interest, give sources of data and details of methods of assessment (measurement). Describe comparability of assessment methods if there is more than one group | 4 & section II of the supplementary file |
| Bias | 9 | Describe any efforts to address potential sources of bias | 4-5 |
| Study size | 10 | Explain how the study size was arrived at | 4 |
| Quantitative variables | 11 | Explain how quantitative variables were handled in the analyses. If applicable, describe which groupings were chosen and why | 4-5, Section III & IV of supplementary file |
| Statistical methods | 12 | (*a*) Describe all statistical methods, including those used to control for confounding | 4-5, Table S4, S5, Section IV of supplementary file |
|  |  | (*b*) Describe any methods used to examine subgroups and interactions | 4-5, Section III & IV of supplementary file |
|  |  | (*c*) Explain how missing data were addressed | 4 |
|  |  | (*d*) If applicable, describe analytical methods taking account of sampling strategy | NA |
|  |  | (*e*) Describe any sensitivity analyses | NA |
| Results | | | |
| Participants | 13* | (a) Report numbers of individuals at each stage of study—eg numbers potentially eligible, examined for eligibility, confirmed eligible, included in the study, completing follow-up, and analysed | 5-6 |
|  |  | (b) Give reasons for non-participation at each stage | 5 |
|  |  | (c) Consider use of a flow diagram | NA |
| Descriptive data | 14* | (a) Give characteristics of study participants (eg demographic, clinical, social) and information on exposures and potential confounders | 6, Table 1, Table S2&S3 |
|  |  | (b) Indicate number of participants with missing data for each variable of interest | Table 1&2, Table S2-S10 |
| Outcome data | 15* | Report numbers of outcome events or summary measures | 5-6, Table S4-S6 |
| Main results | 16 | (*a*) Give unadjusted estimates and, if applicable, confounder-adjusted estimates and their precision (eg, 95% confidence interval). Make clear which confounders were adjusted for and why they were included | NA |
|  |  | (*b*) Report category boundaries when continuous variables were categorized | 5-7 |
|  |  | (*c*) If relevant, consider translating estimates of relative risk into absolute risk for a meaningful time period | NA |
| Other analyses | 17 | Report other analyses done—eg analyses of subgroups and interactions, and sensitivity analyses | Section IV of supplementary file |
| Discussion | | | |
| Key results | 18 | Summarise key results with reference to study objectives | 9-11 |
| Limitations | 19 | Discuss limitations of the study, taking into account sources of potential bias or imprecision. Discuss both direction and magnitude of any potential bias | 12 |
| Interpretation | 20 | Give a cautious overall interpretation of results considering objectives, limitations, multiplicity of analyses, results from similar studies, and other relevant evidence | 9-12 |
| Generalisability | 21 | Discuss the generalisability (external validity) of the study results | 12 |
| Other information | | | |
| Funding | 22 | Give the source of funding and the role of the funders for the present study and, if applicable, for the original study on which the present article is based | 12 |

*Give information separately for exposed and unexposed groups.

## **Table S2: Distribution of FPS, ATOP, IAT, BAOP, Empathy and SATAQ across countries of residence and gender**

| **Measure** | **Overall** | **Country of residence** | | | | | | **Gender** | | |
| --- | --- | --- | --- | --- | --- | --- | --- | --- | --- | --- |
|  | **N = 514**  **M (SD)** | **Australia**  **n = 110**  **M (SD)** | **Canada**  **n = 82**  **M (SD)** | **USA**  **n = 80**  **M (SD)** | **UK**  **n = 81**  **M (SD)** | **Malaysia**  **n = 79**  **M (SD)** | **India**  **n = 82**  **M (SD)** | **Male**  **n = 196**  **M (SD)** | **Female**  **n = 316**  **M (SD)** | **Non-binary**  **n = 2**  **M (SD)** |
| **FPS** | 2.9 (0.82) | 3.1 (0.74) | 3.1 (0.86) | 2.9 (0.80) | 3.1 (0.80) | 2.9 (0.76) | 2.3 (0.70)** | 2.8(0.80) | 2.9(0.83) | 3.9(0.58) |
| **ATOP** | 62.2 (15.4) | 65.1 (15.9)** | 63.2 (17.0)* | 63.8 (18.19) | 62.0(13.70) | 62.1 (14.4) | 55.5(10.20)** | 61.9(14.32) | 62.2(16.12) | 60(4.24) |
| **IAT** | 0.52 (0.45) | 0.56 (0.43)* | 0.57 (0.47)* | 0.68(0.45)** | 0.45 (0.39) | 0.45(0.49) | 0.36 (0.44)** | 0.48(0.46) | 0.54(0.45) | 0.35(0.05) |
| **BAOP** | 19.7 (7.82) | 18.3(7.75) | 17.2(7.38) | 16.9 (7.76) | 16.5 (7.31) | 15.7(5.71) | 16.6(5.7) | 16.22(6.3) | 17.45(7.42) | 16.0(11.31) |
| **Empathy** | 5.3 (0.98) | 5.4 (1.04) | 5.2 (1.07) | 5.0 (0.88) | 5.22 (1.03) | 5.3 (0.89) | 5.4 (0.90) | 5.11(1.07)** | 5.4(0.91)** | 5.2(0.20) |
| **SATAQ** | 3. 3 (0.62) | 3.3 (0.70) | 3.4 (0.62) | 3.3 (0.56) | 3.2 (0.72) | 3.2 (0.53) | 3.1 (0.51) | 3.3(0.54) | 3.3(0.66) | 3.5(0.0) |
| **P<.001; *P<0.05  FPS= Fat phobia scale; IAT= Implicit Association Test; ATOP= Attitude towards PPP with overweight obesity; BAOP= Beliefs about PPP living with overweight or obesity; SATAQ= Sociocultural Attitudes Toward Appearance Questionnaire | | | | | | | | | | |

## **Table S3: Correlations between FPS, IAT, ATOP, BAOP, Empathy and SATAQ**

|  |  | **1** | **2** | **3** | **4** | **5** | **6** |
| --- | --- | --- | --- | --- | --- | --- | --- |
| **1** | **FPS** | 1.00 |  |  |  |  |  |
| **2** | **ATOP** | -0.07 | 1.00 |  |  |  |  |
| **3** | **IAT** | 0.08 | -0.01 | 1.00 |  |  |  |
| **4** | **BAOP** | -0.14** | 0.40** | -0.11* | 1.00 |  |  |
| **5** | **Empathy** | -0.04 | -0.08 | 0.04 | -0.04 | 1.00 |  |
| **6** | **SATAQ** | 0.24** | -0.31** | 0.07 | -0.15** | 0.23** | 1.00 |
| **P<0.001; *P<0.05  FPS= Fat phobia scale; IAT= Implicit Association Test; ATOP= Attitude towards PPP with overweight obesity; BAOP= Beliefs about PPP living with overweight or obesity; SATAQ= Sociocultural Attitudes Toward Appearance Questionnaire | | | | | | | |

## **Table S4: Hierarchical multiple regression of factors associated with explicit weight bias**

|  |  | Model 1 | | | Model 2 | | | Model 3 | | | Model 4 | | |
| --- | --- | --- | --- | --- | --- | --- | --- | --- | --- | --- | --- | --- | --- |
| Variable |  | **B** | **Beta** | ***p-*value** | **B** | **Beta** | ***p-*value** | **B** | **Beta** | ***p-*value** | **B** | **Beta** | ***p-*value** |
| Constant |  | 2.70 |  | <0.001 | 2.64 |  | <0.001 | 2.97 |  | <0.001 | 2.29 |  | <0.001 |
| Age |  | <0.001 | -0.06 | 0.36 | <0.001 | -0.05 | 0.37 | <0.001 | -0.07 | 0.21 | <0.001 | -0.09 | 0.14 |
| Marital status | Single (reference) |  |  |  |  |  |  |  |  |  |  |  |  |
|  | Married | -0.17 | -0.10 | 0.04* | -0.18 | -0.11 | 0.03* | -0.17 | -0.10 | 0.03* | -0.15 | -0.09 | 0.06 |
|  | Divorced/widowed | -0.07 | -0.03 | 0.53 | -0.09 | -0.04 | 0.43 | -0.09 | -0.04 | 0.42 | -0.06 | -0.03 | 0.59 |
| Educational status | High school or less (reference) |  |  |  |  |  |  |  |  |  |  |  |  |
|  | TVET | 0.14 | 0.07 | 0.18 | 0.14 | 0.07 | 0.18 | 0.14 | 0.07 | 0.16 | 0.15 | 0.08 | 0.14 |
|  | BSc/Associate degree | 0.19 | 0.11 | 0.05 | 0.19 | 0.11 | 0.05 | 0.18 | 0.10 | 0.06 | 0.16 | 0.09 | 0.10 |
|  | Masters and above | 0.18 | 0.08 | 0.14 | 0.18 | 0.08 | 0.13 | 0.19 | 0.09 | 0.11 | 0.15 | 0.07 | 0.21 |
| Ethnicity | Asian (reference) |  |  |  |  |  |  |  |  |  |  |  |  |
|  | White | 0.06 | 0.03 | 0.72 | 0.03 | 0.02 | 0.86 | 0.05 | 0.03 | 0.75 | 0.02 | 0.01 | 0.87 |
|  | Others | 0.12 | 0.04 | 0.51 | 0.10 | 0.03 | 0.60 | 0.09 | 0.03 | 0.64 | 0.03 | 0.01 | 0.86 |
| Country of residence | USA (reference) |  |  |  |  |  |  |  |  |  |  |  |  |
|  | Australia | 0.27 | 0.13 | 0.02* | 0.27 | 0.13 | 0.02* | 0.29 | 0.14 | 0.01* | 0.28 | 0.14 | 0.01***** |
|  | Canada | 0.31 | 0.14 | 0.02* | 0.31 | 0.14 | 0.02* | 0.31 | 0.14 | 0.01* | 0.27 | 0.12 | 0.03***** |
|  | UK | 0.30 | 0.13 | 0.02* | 0.30 | 0.13 | 0.02* | 0.27 | 0.12 | 0.03* | 0.27 | 0.12 | 0.03***** |
|  | Malaysia | 0.21 | 0.09 | 0.29 | 0.20 | 0.09 | 0.30 | 0.18 | 0.08 | 0.35 | 0.15 | 0.07 | 0.42 |
|  | India | -0.45 | -0.20 | 0.02* | -0.45 | -0.20 | 0.02* | -0.46 | -0.21 | 0.02 | -0.45 | -0.20 | 0.02***** |
| Employment | Yes (reference) |  |  |  |  |  |  |  |  |  |  |  |  |
|  | No | 0.35 | 0.21 | <0.001** | 0.35 | 0.21 | <0.001** | 0.34 | 0.20 | <0.001** | 0.33 | 0.20 | <0.001****** |
| BMI |  |  |  |  | 0.01 | 0.04 | 0.47 | 0.01 | 0.05 | 0.37 | 0.01 | 0.05 | 0.33 |
| Self-perception of living with obesity | Yes (reference) |  |  |  |  |  |  |  |  |  |  |  |  |
|  | No |  |  |  | -0.07 | -0.04 | 0.42 | -0.09 | -0.05 | 0.29 | -0.04 | -0.02 | 0.67 |
| BAOP |  |  |  |  |  |  |  | -0.02 | -0.16 | <0.001 | -0.02 | -0.13 | <0.001****** |
| SATAQ |  |  |  |  |  |  |  |  |  |  | 0.03 | 0.15 | <0.001****** |
| R^2^ |  | 0.17 |  |  | 0.18 |  |  | 0.20 |  |  | 0.22 |  |  |
| F |  | 7.36****** |  |  | 6.55****** |  |  | 7.18****** |  |  | 7.63****** |  |  |
| R^2^ change |  | 0.17 |  |  | 0.004 |  |  | 0.02 |  |  | 0.02 |  |  |
| **P<0.001; *P<0.05 | | | | | | | | | | | | | |
| TVET: Technical Vocational Education and Training | | | | | | | | | | | | | |
| Others: Black, Multiracial, Hispanic, Indigenous, Middle Eastern | | | | | | | | | | | | | |

## **Table S5: Hierarchical multiple regression of factors associated with attitude towards PPP women living with overweight or obesity**

|  |  | Model 1 | | | Model 2 | | | Model 3 | | | Model 4 | | |
| --- | --- | --- | --- | --- | --- | --- | --- | --- | --- | --- | --- | --- | --- |
| Variable |  | **B** | **Beta** | ***p-*value** | **B** | **Beta** | ***p-*value** | **B** | **Beta** | ***p-*value** | **B** | **Beta** | ***p-*value** |
| Constant |  | 60.35 |  | <0.001 | 51.82 |  | <0.001 | 37.73 |  | <0.001 | 61.68 |  | <0.001 |
| Age |  | 0.02 | 0.03 | 0.65 | 0.02 | 0.02 | 0.72 | 0.07 | 0.08 | 0.15 | 0.09 | 0.10 | 0.04* |
| Marital status | Single (reference) |  |  |  |  |  |  |  |  |  |  |  |  |
|  | Married | 0.69 | 0.02 | 0.66 | 0.32 | 0.01 | 0.84 | -0.19 | -0.01 | 0.90 | -0.86 | -0.03 | 0.53 |
|  | Divorced/widowed | 0.91 | 0.02 | 0.69 | 0.14 | 0.00 | 0.95 | -0.01 | 0.00 | 1.00 | -1.41 | -0.03 | 0.48 |
| Ethnicity | Asian (reference) |  |  |  |  |  |  |  |  |  |  |  |  |
|  | White | 2.23 | 0.07 | 0.47 | 1.06 | 0.03 | 0.73 | -0.16 | 0.00 | 0.95 | 0.55 | 0.02 | 0.84 |
|  | Others | -5.18 | -0.08 | 0.16 | -6.33 | -0.10 | 0.08 | -5.92 | -0.09 | 0.08 | -3.87 | -0.06 | 0.22 |
| Country of residence | USA (reference) |  |  |  |  |  |  |  |  |  |  |  |  |
|  | Australia | 1.25 | 0.03 | 0.58 | 1.18 | 0.03 | 0.59 | 0.08 | 0.00 | 0.97 | 0.32 | 0.01 | 0.87 |
|  | Canada | -0.34 | -0.01 | 0.89 | -0.25 | -0.01 | 0.92 | -0.46 | -0.01 | 0.84 | 0.53 | 0.01 | 0.80 |
|  | UK | -1.42 | -0.03 | 0.57 | -1.47 | -0.03 | 0.55 | -0.58 | -0.01 | 0.80 | -0.84 | -0.02 | 0.70 |
|  | Malaysia | 0.54 | 0.01 | 0.89 | 0.42 | 0.01 | 0.91 | 1.16 | 0.03 | 0.74 | 1.79 | 0.04 | 0.58 |
|  | India | -5.74 | -0.14 | 0.13 | -5.52 | -0.13 | 0.14 | -5.23 | -0.21 | 0.13 | -5.54 | -0.13 | 0.09 |
| BMI |  |  |  |  | 0.37 | 0.14 | 0.01* | 0.33 | 0.12 | 0.01* | 0.30 | 0.11 | 0.02* |
| Self-perception of living with obesity | Yes |  |  |  | 1.03 | 0.03 | 0.54 | 0.11 | 0.00 | 0.94 | 2.16 | 0.07 | 0.15 |
|  | No (reference) |  |  |  |  |  |  |  |  |  |  |  |  |
| BAOP |  |  |  |  |  |  |  | 0.83 | 0.38 | <0.001* | 0.73 | 0.33 | <0.001****** |
| SATAQ |  |  |  |  |  |  |  |  |  |  | -1.20 | -0.30 | <0.001****** |
| R^2^ |  | 0.06 |  |  | 0.08 |  |  | 0.22 |  |  | 0.29 |  |  |
| F |  | 0.06 |  |  | 0.02 |  |  | 0.14 |  |  | 0.08 |  |  |
| R^2^ change |  | 2.95 |  |  | 3.56 |  |  | 10.63 |  |  | 15.08 |  |  |
| **P<0.001; *P<0.05 |  |  |  |  |  |  |  |  |  |  |  |  |  |
| Others: Black, Multiracial, Hispanic, Indigenous, Middle Eastern | | | | | | | | | | | | | |

## **Table S6: Hierarchical multiple regression of factors associated with implicit weight bias**

|  |  | Model 1 | | | Model 2 | | |
| --- | --- | --- | --- | --- | --- | --- | --- |
| Variable |  | **B** | **Beta** | ***p-*value** | **B** | **Beta** | ***p-*value** |
| Constant |  | 0.21 |  | 0.01 | 0.34 |  | 0.02 |
| Age |  | 0.01 | 0.30 | <0.001** | 0.01 | 0.28 | <0.001** |
| Marital status | Single (reference) |  |  |  |  |  |  |
|  | Married | -0.07 | -0.08 | 0.13 | -0.07 | -0.07 | 0.15 |
|  | Divorced/widowed | 0.02 | 0.01 | 0.79 | 0.02 | 0.02 | 0.76 |
| Educational status | High school or less (reference) |  |  |  |  |  |  |
|  | TVET | -0.01 | -0.01 | 0.92 | -0.00 | -0.00 | 0.94 |
|  | BSc/Associate degree | -0.03 | -0.04 | 0.53 | -0.04 | -0.04 | 0.48 |
|  | Masters and above | -0.20 | -0.17 | <0.001** | -0.20 | -0.17 | <0.001** |
| Ethnicity | Asian (reference) |  |  |  |  |  |  |
|  | White | 0.17 | 0.18 | 0.05 | 0.18 | 0.20 | 0.04* |
|  | Others | 0.19 | 0.10 | 0.07 | 0.19 | 0.10 | 0.07 |
| Country of residence | USA (reference) |  |  |  |  |  |  |
|  | Australia | -0.14 | -0.13 | 0.03* | -0.14 | -0.12 | 0.04* |
|  | Canada | -0.08 | -0.07 | 0.24 | -0.09 | -0.07 | 0.24 |
|  | UK | -0.15 | -0.12 | 0.04* | -0.16 | -0.13 | 0.02* |
|  | Malaysia | 0.06 | 0.04 | 0.61 | 0.05 | 0.04 | 0.67 |
|  | India | 0.07 | 0.05 | 0.54 | 0.06 | 0.05 | 0.57 |
| Employment | Yes (reference) |  |  |  |  |  |  |
|  | No | -0.10 | -0.11 | 0.04* | -0.10 | -0.11 | 0.03* |
| BAOP |  |  |  |  | -0.01 | -0.11 | 0.01* |
| R^2^ |  | 0.13 |  |  | 0.14 |  |  |
| F |  | 0.13 |  |  | 0.01 |  |  |
| R^2^ change |  | 5.3 |  |  | 5.43 |  |  |
| **P<0.001; *P<0.05 | | | | | | | |
| Others: Black, Multiracial, Hispanic, Indigenous, Middle Eastern | | | | | | | |

# **Section II: Description of the measures**

*Explicit weight bias*: Explicit weight bias was assessed by the Fat Phobia Scale (FPS)[1], a validated 14-item Likert questionnaire assessing beliefs and feelings towards people living with overweight or obesity. Possible scores ranged from one to five, with high scores indicating more “fat phobia” [1]. Cronbach’s alpha was 0.92.

*Attitude towards PPP living with obesity:* Attitude towards PPP women with obesity was assessed using the Attitudes Towards People with Obesity (ATOP) tool, a 20-item questionnaire measuring stereotypical attitudes about persons with obesity, inclusive of perceptions about their self-esteem, personality, and social quality of life [2]. Participants indicated their agreement (I strongly disagree to I strongly agree) with the statements presented along a six-point Likert scale. Possible scores ranged from zero to 120. High scores demonstrate more positive attitudes toward persons with obesity [2]. Cronbach’s alpha was 0.77.

*Implicit weight bias*: Implicit weight bias was assessed using a Weight Implicit Association Test (IAT), [3] a computer-based reaction-time task that uses response latencies to measure the relative strength of an association between two concept categories. The weight IAT compares the time required to categorise images of people living with obesity and “thin” people together with positive and negative words. The IAT difference scores were categorised according to commonly used cut points for slight (d score= ≥0.15 and <0.35), moderate (d score= ≥0.35 and <0.65), and strong bias (d score ≥0.65) [3]. The IAT was hosted by Project Implicit, a non-profit organisation that measures implicit biases and attitudes against various social groups. The IAT test was linked with the Qualtrics survey and the participants were able to access it as part of the main survey. Project implicit provided services such as maintenance, storage, secure data exchange, coding information, and data checking.

**Independent variables**

*Controllability beliefs*: Controllability beliefs of obesity was assessed using the Beliefs About Persons Living with Obesity Scale (BAOP), a validated, eight-item questionnaire measuring beliefs surrounding the causes and controllability of obesity [2]. Participants indicated their agreement (I strongly disagree to I strongly agree) with the statements presented along a six-point Likert-scale. Possible scores ranged from 0-48 with higher scores indicating a less biased belief [2]. Cronbach’s alpha was 0.66.

*Societal norms*: The Awareness sub-scale of the Sociocultural Attitudes Toward Appearance Questionnaire (SATAQ) was used to assess societal norms regarding perceptions towards people with larger body sizes.[4] The SATAQ measures people’s awareness and internalisation of sociocultural standards or norms regarding body appearance and size. Items were rated on a five-point Likert-type scale (completely disagree to completely agree), with higher scores representing higher levels of awareness of sociocultural standards of thinness ideals [4]. Cronbach’s alpha was 0.50.

*Empathy*: The Empathy subscale of the Fat Attitudes Assessment Toolkit (FAAT) was used to assess the degree to which respondents recognise and empathise with the negative evaluations that women living with overweight or obesity face in everyday life and the impact of these evaluations [5]. This subscale contains seven items that are scored on a seven-point Likert scale (Strongly disagree to Strongly agree). Higher scores indicate higher levels of empathy evaluations [5]. Cronbach’s alpha was 0.85.

*Sociodemographic characteristics:* Participants completed questions about their country of residence, age, gender, race, marital status, employment status, educational status, height, weight and weight self-perception. Participants’ BMI was calculated from self-reported height and weight. The BMI was categorised according to World Health Organisation recommendations [6].

# **Section III: Latent profile analysis**

## **Table S7. Model fit indices for Latent Profiles**

|  | -2LL | AIC | BIC | aBIC | VLMR | LMR | BLRT |
| --- | --- | --- | --- | --- | --- | --- | --- |
| 1 Profile | 8374.192 | 8388.192 | 8417.888 | 8395.668 | - | - | - |
| 2 Profiles | 8066.99 | 8096.989 | 8160.622 | 8113.010 | *p* < 0.001 | *p* < 0.001 | *p* < 0.001 |
| 3 Profiles | 8019.852 | 8065.853 | 8163.424 | 8090.418 | *p* = 0.1221 | *p* = 0.1264 | *p* < 0.001 |
| 4 Profiles | 7975.418 | 8037.418 | 8168.927 | 8070.528 | *p* = 0.0028 | *p* = 0.0031 | *p* < 0.001 |
| -2LL = -2 Log Likelihood; AIC = Akaike Information Criteria; BIC = Bayesian Information Criteria; aBIC = sample size adjusted Bayesian Information Criteria; VLMR = Vuong-Lo-Mendell Rubin Likelihood Ratio Test ; LMR = Lo-Mendell-Rubin Likelihood Ratio Test; BLRT = Bootstrapped Likelihood Ratio test | | | | | | | |

# **Section IV: Univariate analysis results**

## **Table S8: Univariate analysis of factors associated with explicit weight bias (FPS)**

| Variable | B | β | P- value |
| --- | --- | --- | --- |
| Age | 0.007 | 0.144 | 0.002* |
| Gender F (ref: M) | 0.057 | 0.034 | 0.444 |
| Marital status | | | |
| Single (ref) | | | |
| Married | -0.19 | -0.116 | 0.02* |
| Divorced/widowed | 0.055 | 0.025 | 0.62 |
| Educational status | | | |
| High School or less (ref) |  |  |  |
| Technical or vocational training | 0.150 | 0.075 | 0.163 |
| Bachelor/Associate degree | -0.024 | -0.014 | 0.802 |
| Master's degree and above | -0.289 | -0.136 | 0.01* |
| Ethnicity | | | |
| Asian(ref) | | | |
| White | 0.387 | 0.23 | 0.001* |
| Others | 0.407 | 0.119 | 0.009* |
| Country of residence | | | |
| USA (ref) | | | |
| Australia | 0.198 | 0.099 | 0.083 |
| Canada | 0.243 | 0.108 | 0.047* |
| UK | 0.211 | 0.094 | 0.084 |
| Malaysia | 0.018 | 0.008 | 0.883 |
| India | -0.604 | -0.269 | 0.001* |
| Employment | | | |
| Yes (ref) | | | |
| No | 0.415 | 0.251 | 0.001* |
| BMI | 0.018 | 0.128 | 0.004* |
| Self-perception of living with obesity | | | |
| Yes | 0.16 | 0.092 | 0.03* |
| No (ref) | | | |
| Family member living with overweight or obesity | | | |
| Yes | 0.068 | 0.041 | 0.355 |
| No (ref) | | | |
| Friend living with overweight or obesity | | | |
| Yes | -0.048 | -0.029 | 0.512 |
| No (ref) | | | |
| BAOP | -0.016 | -0.141 | 0.001* |
| Empathy | -0.004 | -0.031 | 0.397 |
| SATAQ | 0.051 | 0.237 | 0.001* |
| **p* < 0.05  Note: Small group removed for gender: Nonbinary (n =2) | | | |

## **Table S9: Univariate analysis of factors associated with attitude towards PPP living with overweight or obesity (ATOP)**

| Variable | B | β | P- value |
| --- | --- | --- | --- |
| Age | 0.124 | 0.142 | 0.001* |
| Gender F (ref: M) | 0.375 | 0.012 | 0.788 |
| Marital status |  |  |  |
| Single(ref) |  |  |  |
| Married | 1.292 | 0.042 | 0.398 |
| Divorced | 4.136 | 0.099 | 0.046* |
| Educational status |  |  |  |
| High School or less (ref) |  |  |  |
| Technical or vocational training | -1.019 | -0.027 | 0.617 |
| Bachelor/Associate degree | -1.926 | -0.06 | 0.284 |
| Master's degree and above | -1.697 | -0.043 | 0.423 |
| Ethnicity |  |  |  |
| Asian(ref) | | | |
| White | 5.089 | 0.162 | 0.001* |
| Others | -3.712 | -0.058 | 0.204 |
| Country of residence |  |  |  |
| USA (ref) | | | |
| Australia | 1.328 | 0.035 | 0.551 |
| Canada | -0.619 | -0.015 | 0.795 |
| UK | -1.838 | -0.044 | 0.442 |
| Malaysia | -1.673 | -0.039 | 0.487 |
| India | -8.314 | -0.198 | 0.001* |
| Employment | | | |
| Yes (ref) | | | |
| No | 2.08 | 0.067 | 0.129 |
| BMI | 0.5 | 0.19 | 0.001* |
| Self-perception of living with obesity | | | |
| Yes | 4.167 | 0.13 | 0.003* |
| No (ref) | | | |
| Family member living with overweight or obesity | | | |
| Yes | 1.316 | 0.043 | 0.337 |
| No (ref) | | | |
| Friend living with overweight or obesity | | | |
| Yes | 0.786 | 0.025 | 0.565 |
| No (ref) | | | |
| BAOP | 0.869 | 0.398 | 0.001* |
| Empathy | -0.169 | -0.076 | 0.087 |
| SATAQ | -1.188 | -0.295 | 0.001* |
| **p* < 0.05  Note: Small group removed for gender: Nonbinary (n =2) | | | |

## **Table S10: Univariate analysis of factors associated with implicit weight bias (IAT)**

| Variable | B | β | P- value |
| --- | --- | --- | --- |
| Age | 0.007 | 0.283 | 0.001* |
| Gender F (ref: M) | 0.058 | 0.062 | 0.162 |
| Marital status |  |  |  |
| Single (ref) |  |  |  |
| Married | -0.035 | -0.038 | 0.441 |
| Divorced | 0.18 | 0.145 | 0.003* |
| Educational status |  |  |  |
| High School or less (ref) |  |  |  |
| Technical or vocational training | 0.012 | 0.011 | 0.839 |
| Bachelor/Associate degree | -0.044 | -0.046 | 0.409 |
| Master's degree and above | -0.226 | -0.191 | 0.001* |
| Ethnicity | | | |
| Asian(ref) | | | |
| White | 0.194 | 0.207 | 0.001* |
| Others | 0.138 | 0.073 | 0.111 |
| Country of residence | | | |
| USA (ref) | | | |
| Australia | -0.117 | -0.105 | 0.076 |
| Canada | -0.103 | -0.082 | 0.145 |
| UK | -0.225 | -0.179 | 0.002* |
| Malaysia | -0.222 | -0.175 | 0.002* |
| India | -0.318 | -0.254 | 0.001* |
| Employment |  |  |  |
| Yes (ref) | | | |
| No | 0.093 | 0.101 | 0.023* |
| BMI | -0.006 | -0.075 | 0.092 |
| Self-perception of living with obesity | | | |
| Yes | -0.063 | -0.066 | 0.135 |
| No (ref) |  |  |  |
| Family member living with overweight or obesity | | | |
| Yes | -0.075 | -0.081 | 0.066 |
| No (ref) | | | |
| Friend living with overweight or obesity | | | |
| Yes | 0.021 | 0.023 | 0.607 |
| No (ref) | | | |
| BAOP | -0.007 | -0.112 | 0.011* |
| Empathy | -0.002 | -0.032 | 0.378 |
| SATAQ | 0.007 | 0.057 | 0.20 |
| **p* < 0.05  Note: Small group removed for gender: Nonbinary (n =2) | | | |

# **References**

1. Bacon JG, Scheltema KE, Robinson BE. Fat phobia scale revisited: the short form. Int J Obes Relat Metab Disord. 2001;25(2):252-7.

2. Allison DB, Basile VC, Yuker HE. measurement of attitudes toward and beliefs about obese persons. Int J Eat Disord. 1991;10(5):599-607.

3. Greenwald AG, Poehlman TA, Uhlmann EL, Banaji MR. Understanding and Using the Implicit Association Test: III. Meta-Analysis of Predictive Validity. J Pers Soc Psychol. 2009;97(1):17-41.

4. Heinberg LJ, Thompson JK, Stormer S. Development and validation of the sociocultural attitudes towards appearance questionnaire. Int J Eat Disord. 1995;17(1):81-9.

5. Cain P, Donaghue N, Ditchburn G. Development and validation of the Fat Attitudes Assessment Toolkit (FAAT): A multidimensional nonstigmatizing measure of contemporary attitudes toward fatness and fat people. Journal of applied social psychology. 2022;52(12):1121-45.

6. Organization WH. Obesity - Preventing and Managing the Global Epidemic: Report on a WHO Consultation. 1 ed. London: World Health Organization; 2000. xii-xii p.

7. Muthén LK, Muthén BO. Mplus User’s Guide. Eighth Edition1998-2018.

8. Nylund KL, Asparouhov T, Muthén BO. Deciding on the Number of Classes in Latent Class Analysis and Growth Mixture Modeling: A Monte Carlo Simulation Study. Structural equation modeling. 2007;14(4):535-69.

9. Nylund-Gibson K, Grimm R, Quirk M, Furlong M. A Latent Transition Mixture Model Using the Three-Step Specification. Structural equation modeling. 2014;21(3):439-54.

10. Asparouhov T, Muthén B. Auxiliary Variables in Mixture Modeling: Three-Step Approaches Using M plus. Structural equation modeling. 2014;21(3):329-41.
